# The Role of Antigen Carbohydrate 125 in Modulating Soluble ST2: Prognostic-Related Effects in Acute Heart Failure

**DOI:** 10.3390/biom15040602

**Published:** 2025-04-18

**Authors:** Arancha Martí-Martínez, Julio Núñez, Herminio López-Escribano, Elena Revuelta-López, Anna Mollar, Marta Peiró, Juan Sanchis, Antoni Bayés-Genís, Arturo Carratala, Òscar Miró, Pere Llorens, Pablo Herrero-Puente

**Affiliations:** 1 Clinical Biochemistry Department, Valencia Clinical Hospital, 46010 Valencia, Spain; aranchamartimartinez@gmail.com (A.M.-M.); lopez_heresc@gva.es (H.L.-E.); carratala_art@gva.es (A.C.); 2 Cardiology Department, Valencia Clinical Hospital, Instituto de Investigación Sanitaria (INCLIVA), 46010 Valencia, Spain; annammollar@gmail.com (A.M.); peiro_marsig@gva.es (M.P.); sanchis_juafor@gva.es (J.S.); 3 Centro de Investigación Biomédica en Red Enfermedades Cardiovasculares, 28022 Madrid, Spain; erevuelta@igtp.cat (E.R.-L.); abayesgenis@gmail.com (A.B.-G.); 4 Cardiology Department, Hospital Universitari Germans Trias i Pujol, Universitat Autònoma de Barcelona, 08916 Badalona, Spain; 5 Emergency Department, Hospital Clínic, Instituto de Investigaciones Biomédicas August Pi i Sunyer (IDIBAPS), University of Barcelona, 08036 Barcelona, Spain; omiro@clinic.cat; 6 Emergency Department, General University Hospital of Alicante, 03010 Alicante, Spain; llorens_ped@gva.es; 7 Emergency Department, Asturias Central University Hospital, 33011 Oviedo, Spain

**Keywords:** acute heart failure, CA125, sST2, inflammatory modulator

## Abstract

Background: Acute heart failure (AHF) is a complex syndrome associated with high mortality and hospital readmissions, characterized by volume overload and inflammation. Soluble ST2 (sST2) and antigen carbohydrate 125 (CA125) are emerging biomarkers that reflect these processes and may interact to influence long-term outcomes in AHF patients. This study aims to examine the prognostic relationship between sST2 and CA125 in predicting mortality and heart failure (HF)-related hospitalizations in patients with decompensated heart failure. Methods: In a cohort of 635 patients with AHF, we investigated whether the prognostic value of sST2 varies according to CA125 levels (≤35 vs. >35 U/mL). The endpoints were: (a) time to all-cause death, and (b) the combination of time to death or new HF admission. Results: This study of EAHFE registry data shows that the association between sST2 and long-term adverse outcomes (mortality and HF hospitalizations) in patients with AHF was differentially influenced by CA125 concentrations (*p*-value for interactions = 0.031 and 0.029, respectively). Higher sST2 was associated with the risk of death and the combined risk of death/HF readmission when CA125 was >35 U/mL [HR = 1.02 (CI 95%: 1.01–1.04), *p* = 0.006 and 1.02 (CI 95%: 1.01–1.03); *p* = 0.013 per increase in 10 ng/mL, respectively], but not when CA125 was ≤35 U/mL. Conclusions: This study highlights the prognostic interaction between sST2 and CA125 in AHF. Elevated sST2 predicts poor outcomes mainly in patients with high CA125 levels (>35 U/mL), suggesting CA125’s role in modulating inflammatory activity in HF. Further research is needed.

## 1. Introduction

Acute heart failure (AHF) is a heterogeneous clinical syndrome associated with high mortality rates and frequent hospital readmissions, constituting a significant burden on healthcare systems both medically and economically [1,2]. One of its defining characteristics is volume overload combined with systemic inflammation, wherein soluble Suppression of Tumorigenicity 2 (sST2) serves as a crucial indicator of inflammatory and fibrotic processes [3]. Elevated plasma levels of sST2 in AHF patients have been closely linked to increased disease severity, poor diuretic response, and a heightened risk of cardiovascular mortality [4].

Beyond sST2, antigen carbohydrate 125 (CA125), also known as mucin 16 (MUC16), has emerged as another promising biomarker in AHF, particularly in reflecting volume overload and inflammation [5]. Recent research suggests that CA125 may play a causal role as a ligand, actively modulating inflammatory responses through interactions with various molecular targets. Notably, CA125 has been identified as a binding partner for soluble lectins, such as galectin-1 (Gal-1) and Gal-3 [6]. These interactions with glycosylated proteins are known to influence the biological activity of galectins. In the context of AHF, previous studies have shown that the prognostic impact of Gal-3 is modulated by CA125 levels. Specifically, elevated Gal-3 is linked to worse outcomes in patients with high CA125 concentration, whereas this association is not observed in those with lower CA125 levels, highlighting a potential interplay between these biomarkers [7].

Structurally, MUC16 has a highly glycosylated extracellular domain that enables interactions with other proteins, allowing it to function as a modulator in various biological processes [6]. Similarly, sST2 contains an immunoglobulin-like domain in its extracellular region, conferring structural flexibility that may facilitate interactions with proteins beyond its native interleukin-33 (IL-33) receptor [8]. Following this line of thought, we recently found an interaction between CA125 and sST2 in a small sample of patients with AHF and renal dysfunction [9]. Specifically, elevated sST2 was significantly associated with an increased risk of cardiovascular (CV)–renal hospitalizations only in patients with CA125 >35 U/mL but not when CA125 was ≤35 U/mL. However, this prior observation was restricted to a selected population with kidney dysfunction on admission. Additionally, the small sample size precluded formulation of robust estimates in terms of mortality risk.

In the current study, we wanted to confirm whether the prognostic interaction found between CA125 and sST2 is also replicated in a larger, non-selected sample of patients with decompensated heart failure in an emergency room. Thus, we aimed to examine the association between sST2 and long-term adverse clinical outcomes (mortality and heart failure (HF) hospitalizations) across CA125 status.

## 2. Materials and Methods

### 2.1. Study Sample

We analyzed data from 635 consecutive patients with AHF included in the Epidemiology of Acute Heart Failure in Spanish Emergency Departments (EAHFE) registry, in which sST2 and CA125 levels were measured. The EAHFE registry is a multicenter, non-interventional, analytical cohort study with prospective follow-up [10,11]. It includes patients diagnosed with AHF who were enrolled across multiple Spanish hospital emergency departments (EDs). The registry systematically collects comprehensive data on clinical characteristics, laboratory findings, therapeutic interventions, and outcomes in AHF patients. To date, the EAHFE registry has completed eight phases of patient inclusion. For the present study, cases were randomly selected.

The participating hospitals are distributed throughout Spain and include a diverse mix of university, referral, and community hospitals. The EDs consistently enroll all consecutive patients treated for AHF. The diagnostic criteria for AHF are based on the presence of typical symptoms (e.g., dyspnea, orthopnea, paroxysmal nocturnal dyspnea), acute clinical signs of AHF (e.g., third heart sound, pulmonary crackles, jugular venous distension >4 cm, resting sinus tachycardia, peripheral edema, hepatomegaly, or hepatojugular reflux), and radiological evidence of pulmonary congestion. The only exclusion criterion is the presence of ST-elevation myocardial infarction as the primary diagnosis with associated AHF. At each hospital, a training meeting was held to standardize inclusion and exclusion criteria, and any doubts were reviewed by the principal investigator of each center. This protocol has been applied consistently across all eight recruitment phases of the registry, with minimal changes to the collected variables.

The EAHFE registry adheres to the ethical principles outlined in the Declaration of Helsinki for medical research involving human subjects. All patients provided written informed consent for participation in the registry. 

The study was approved by the Institutional Review Board (or Ethics Committee) of 385 Hospital Clínico Universitario de Valencia (protocol code 2024/371) approved 3 March 2025.

### 2.2. Biomarkers Assessment

Plasma samples were collected in the first blood sample obtained at the ED of the participating hospitals, upon patient arrival. All samples were collected in lithium heparin tubes, processed and stored at −20 °C until analysis. CA125 and sST2 biomarkers were processed at the Clinical Biochemistry and Molecular Pathology Laboratory of the University Clinical Hospital of Valencia.

The commercially available assays used were microparticle chemiluminescent immunoassay Alinity-i CA125 II (Abbott^®^, Abbott Park, IL, USA) and turbidimetric immunoassay SEQUENT-IATM ST2 (Critical Diagnostics^®^, San Diego, CA, USA), adapted for the Alinity-c analyzer (Abbott^®^). The mean values of the quality control for CA125 were as follows: Level 1 = 22 U/mL, Level 2 = 37 U/mL and Level 3 = 74 U/mL, while for sST2, they were the following: Level 1 = 18 ng/mL and Level 2 = 54 ng/mL. The total variation coefficients for each assay, as indicated by the manufacturers, were 4.2%, 3.7%, and 2.7% for the low (39.3 U/mL), medium (271.2 U/mL) and high (570.7 U/mL) concentrations of CA125 and 8.6%, 3.8%, and 2.2% for the low (25.7 ng/mL), medium (75.4 ng/mL) and high (160.6 ng/mL) concentrations of sST2, respectively. Both biomarkers were categorized based on established cut-off values reported in the literature (35 U/mL and ng/mL, respectively).

### 2.3. Endpoints

In the current analysis, we aimed to assess the relationship between exposures and (a) time to all-cause death, and (b) the combined time to death or new HF admission (excluding the index episode).

### 2.4. Statistical Analysis

Continuous variables were reported as mean ± standard deviation (SD) or median [interquartile range (IQR)] when appropriate. sST2 and CA125 were categorized based on established threshold levels widely used in the literature and the technical assays (sST2: ≤35 vs. >35 ng/mL and CA125: ≤35 vs. >35 U/mL) [7,9]. Group differences for sST2 and CA125 were analyzed with ANOVA or Kruskal–Wallis rank test, as appropriate. Discrete variables were expressed as percentages and compared using the χ^2^ test. Correlation between CA125 and sST2 were assessed by Spearman correlation index. Mortality and new HF admission rates were reported as the number of events per 10 person-years (P-Y). The associations between the exposures (CA125 and sST2) and adverse clinical events were examined by multivariate Cox regression analyses. Specifically, we assessed whether the risk of sST2 along the continuum was statistically modified by CA125 strata (>35 U/mL vs. ≤35 U/mL).

Candidate covariates for multivariable analysis were selected based on prior literature, biological plausibility, and clinical relevance, irrespective of their statistical significance in univariable analyses. To mitigate model overfitting and improve parsimony, a backward stepwise selection procedure was employed, applying a liberal retention threshold (*p* < 0.20). Signs and symptoms of volume overload were forcibly retained in the final model regardless of statistical significance in order to account for potential unmeasured confounding related to clinical congestion.

The final models for the endpoints included the following covariates: age, sex, prior New York Heart Association (NYHA) class under stable conditions, history of HF, history of ischemic heart disease, atrial fibrillation at admission, systolic blood pressure, heart rate, Barthel index score, orthopnea, paroxysmal nocturnal dyspnea, third heart sound, jugular venous distension, pleural effusion, peripheral edema, hemoglobin, and creatinine levels. We performed a sensitivity analysis adjusting for MEESSI risk score when it was available (*n* = 406 patients). This score provides an accurate risk stratification in patients with AHF in EDs [12]. A two-sided *p*-value of <0.05 was considered the threshold for statistical significance. All analyses were conducted using STATA version 16.1 [Stata Statistical Software, Release 16 (2019); StataCorp LP, College Station, TX, USA].

## 3. Results

### 3.1. Baseline Characteristics

The baseline and biomarker characteristics of the total cohort are detailed in Table 1. The mean ± SD age of the sample was 82.4 ± 10 years, and 339 (53.4%) were women. Most patients showed a prior history of hypertension (84.1%) and a prior history of HF (63.1%). At presentation, the proportion of patients with atrial fibrillation was 49.9% and most of them showed clinical evidence of volume overload (Table 1). The median (*p* 25% to *p* 75%) creatinine, hemoglobin and NT-proBNP were 1.2 mg/dL (0.9–1.6), 12 g/dL (10.6–13.4), and 4207 pg/mL (2280–8421), respectively. Regarding the exposures, the median (*p* 25% to *p* 75%) of CA125 and sST2 were 44 U/mL (19.0–94.0), and 49.2 ng/mL (27.3–88.5), respectively. The proportion of patients with CA125 and sST2 values above 35 was 57.3% and 67.2%, respectively (Table 1). Spearman correlation coefficient showed CA125 and sST2 were weak and positively correlated (r = 0.237, *p* < 0.001).

### 3.2. Baseline Characteristics Across CA125 and sST2 Categories

Among the 284 patients (44.7%) with elevated levels of both CA125 (>35 U/mL) and sST2 (>35 ng/mL), there was a higher prevalence of women, and most had lower systolic blood pressure. They exhibited significantly reduced left ventricular ejection fraction (LVEF) and higher levels of NT-proBNP. This group also had a higher prevalence of pleural effusion and poorer functional status, as indicated by lower Barthel Index scores compared to other groups. In summary, patients with both biomarkers elevated displayed a worse risk profile (Table 2).

### 3.3. Adverse Clinical Events

At a median (*p* 25% to *p* 75%) follow-up of 380 days (126 to 456), we registered 216 (34.0%) all-cause deaths, and 152 (23.9%) new HF admissions. The total of patients that experienced the combined endpoint of death or new HF admission was 295 (46.5%). The annualized rates of death and the combination of death or new HF admissions were 3.9 (CI 95%: 3.5 to 4.5) and 6.6 (CI 95%: 5.9 to 7.4) per 10 P-Y, respectively.

### 3.4. Relationship Between CA125 and sST2 as Main Terms with Adverse Clinical Events

**Figure 1:**
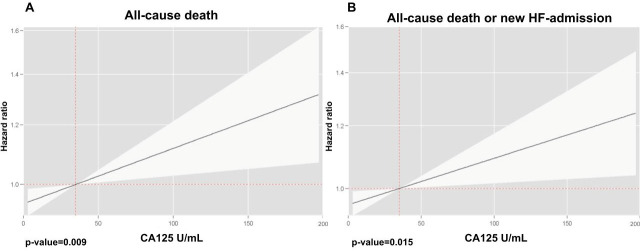
Relationship between CA125 with adverse clinical events. (**A**) All-cause death; (**B**) all-cause death or HF admission.

**Figure 2:**
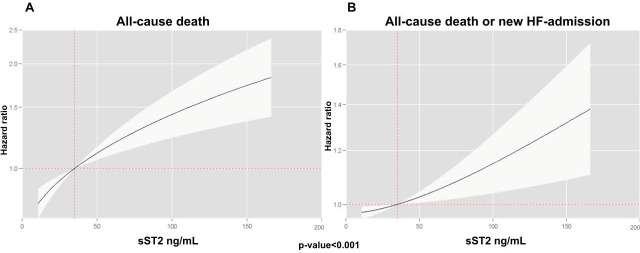
Relationship between sST2 with adverse clinical events. (**A**) All-cause death; (**B**) all-cause death or HF admission.

Multivariate analyses, incorporating both biomarkers into the model, revealed that CA125 and sST2 were independently associated with both endpoints. The risk pattern for CA125 followed a positive and linear trend (Figure 1), while for sST2, the relationship was also positive but exhibited a slightly nonlinear behavior (Figure 2).

### 3.5. The Modifying Prognostic Role of sST2 Across CA125

#### 3.5.1. All Cause-Death

**Figure 3:**
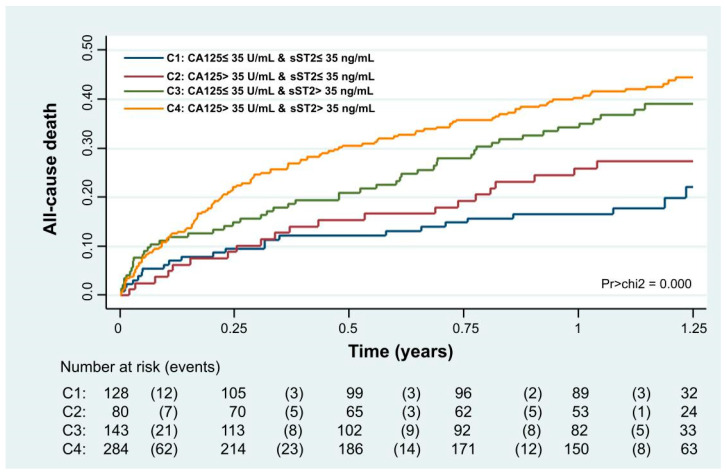
Kaplan-Meier curve depicting the all-cause death rates across CA125 and sST2 categories.

Kaplan-Meier analyses revealed that combining both biomarkers (CA125 > 35 U/mL and sST2 > 35 ng/mL) allowed for a more refined risk stratification. Patients with both biomarkers below the threshold had the lowest mortality risk through the follow-up, those with one elevated biomarker had an intermediate risk, and those with elevated biomarkers had the highest risk (Figure 3, *p* < 0.001).

**Figure 4:**
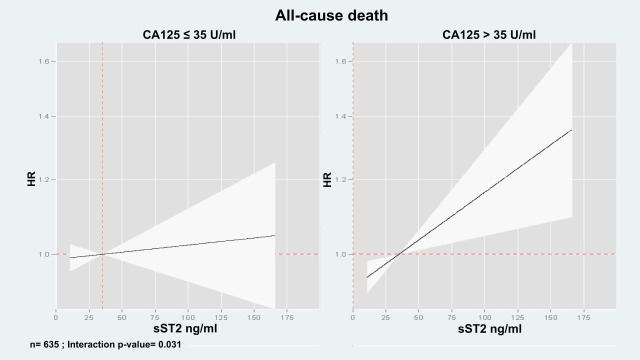
sST2 and its association with the risk of all-cause death expressed as adjusted hazard ratios.

Multivariate analyses examining the association of sST2 with mortality across CA125 status (≤35 U/mL vs. >35 U/mL) revealed a significant interaction (interaction *p*-value = 0.031). In patients with CA125 > 35 U/mL, sST2 values (along their continuum) were positive and linearly associated with mortality risk (Figure 4). Indeed, the HRs [95% CI, (*p*-value)] per increase in 10 ng/mL of sST2 were 1.02 (CI 95%: 1.01 to 1.04, *p* = 0.006). By contrast, in individuals with CA125 ≤ 35 U/mL, sST2 was not related to the risk of death (HR: 1.00, CI 95%: 0.99 to 1.02, *p* = 0.619).

**Figure 5:**
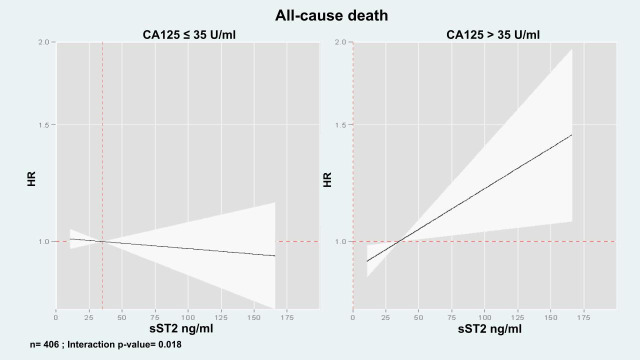
sST2 and its association with the risk of all-cause death expressed as adjusted hazard ratios including the MEESSI score in the analysis.

In a sensitivity analysis, performed in 406 patients in which MEESSI risk score was available, a similar heterogeneous association was found when estimates of risk were adjusted for MEESSI risk score (Figure 5).

#### 3.5.2. Combination of Death or New HF Admission

**Figure 6:**
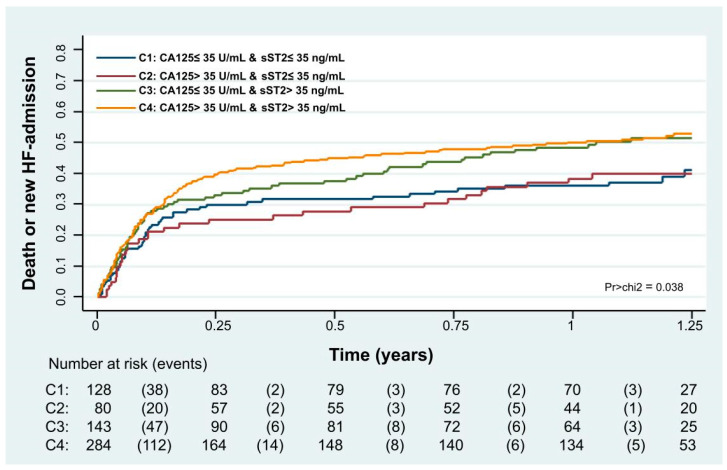
Kaplan–Meier curves depicting the death or new HF-admission rates across CA125 and sST2 categories.

Kaplan–Meier curves also showed that combining both biomarkers, the combination of death or new HF admission risk was higher when both biomarkers were elevated, especially for the first 6 months. After this period, the curves no longer diverged (Figure 6, *p* = 0.038).

**Figure 7:**
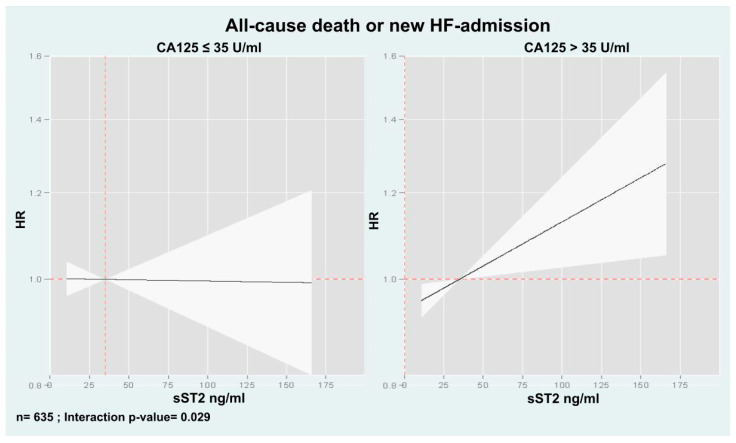
sST2 and its association with the risk of all-cause death or HF admission expressed as adjusted hazard ratios.

After multivariate adjustment, we also found that the sST2 risk was modified based on CA125 >35 U/mL vs. ≤35 U/mL (interaction *p*-value = 0.029). In patients with CA125 >35 U/mL, higher sST2 was significantly associated with an increased risk of the event (HR = 1.02, CI 95%: 1.01 to 1.03, *p* = 0.013, per increase in 10 ng/mL), as shown in Figure 4. Conversely, in patients with CA125 ≤ 35 U/mL, sST2 was not related to this endpoint (Figure 7). Likewise, this heterogeneous risk across CA125 strata was also present when estimates were adjusted for MEESSI risk score.

Covariate risk estimates of the multivariate models are presented in Table A1 and Table A2 in Appendix A.

## 4. Discussion

In the present post-hoc study of the EAHFE registry, we found that the association between sST2 and long-term adverse outcomes (mortality and HF hospitalizations) in patients with AHF was differentially influenced by CA125 concentrations. Indeed, the increased risk attributable to elevated sST2 levels was found when CA125 was >35 U/mL but not in those with CA125 ≤ 35 U/mL. This study builds upon previous findings, where the prognostic value of sST2 and Gal-3 were significantly influenced by CA125 levels [7,9]. The interaction between sST2 and CA125 underscores a complex interplay between inflammation and congestion in AHF, reaffirming the potential role of this mucin in modulating inflammatory and reparative activity in patients with HF [7,9,13]. The underlying biological mechanisms for this interaction merit further exploration.

### 4.1. Structure and Pathophysiology of MUC16 (CA125)

MUC16 is a large transmembrane mucin with three major domains: an extracellular N-terminal domain, a large tandem repeat domain, and a C-terminal domain (CTD), with potential cleavage locations [6,14]. It plays a role in cellular protection, signaling, and tumor progression [7,14]. Cleavage of MUC16 occurs under conditions of cellular homeostasis, tumor progression, and inflammation, which facilitates the release of its N-terminal domain into circulation, making CA125 a valuable biomarker [7]. Meanwhile, after cleavage, the CTD can remain on the cell surface or translocate to the nucleus, binding to chromatin, where it acts as a transcriptional co-regulator [6]. In malignancies such as ovarian [15] and pancreatic cancer [16], MUC16 CTD translocation drives the expression of invasion-related genes, such as NRP2 in pancreatic cancer, promoting disease progression. Moreover, recent evidence has identified MUC16 CTD as a component of a protein-binding complex, mediated by N-glycan components, which includes EGFR, β1 integrin, and Gal-3 on the cell surface [6,17]. These interactions positively regulate epithelial-to-mesenchymal transition (EMT), a process that contributes to cancer metastasis, organ fibrosis, and tissue remodeling [6,18]. EMT is also implicated in pulmonary fibrosis, where TGF-β1 forms a protein complex with MUC16 CTD to activate fibrotic pathways [18]. Similar mechanisms have been observed in prior studies [13], performed in myocardial tissue, where MUC16 expression in epicardial fat correlates with markers of inflammation and fibrosis, suggesting a contributory role in myocardial remodeling. Previous research has linked EMT and soluble inflammation mediators, such as tumor necrosis factor (TNF)-α, IL-6, and IL-1β [17].

### 4.2. Relationship of CA125 with Pro-Inflammatory Pathways and sST2

AHF is not only a consequence of structural or functional damage to the heart, it is also produced by an exacerbated inflammatory response. In this scenario, epicardial adipose tissue shifts its biology to a pro-inflammatory state, becoming a source of several pro-inflammatory cytokines, which have been associated with fibroblast proliferation, collagen synthesis, and myofibroblast activation [13]. Along this line of thought, CA125 is upregulated in most cases of AHF, and in this setting is associated with the severity of volume overload and inflammatory activity [14]. Inflammatory stimuli are identified as a main driver for CA125 synthesis in mesothelial cells [14] and prior studies have reported a positive correlation among cytokines and CA125 in AHF [19] and an association with higher risk of adverse outcomes [20].

The mediator linking volume overload and elevated CA125 levels in AHF is unknown. However, we speculate that the mechanical stress and inflammation caused by excessive tissue fluid accumulation [14] subsequently activates c-Jun N-terminal kinase (JNK) pathways, leading to an increase in CA125 levels [5]. First, JNK activation promotes the synthesis of CA125. Second, changes in cell morphology and membrane stability, along with mechanical stress, activate the O-glycosylated extracellular domain of CA125, facilitating its shedding from mesothelial cells and thereby increasing its concentration in the peripheral circulation [21].

ST2, located on chromosome 2q12 within the IL-1 gene cluster, is expressed primarily as two key isoforms: the membrane-bound ST2 (ST2L) and the soluble ST2, sST2 [22,23,24]. ST2L serves as the receptor for IL-33 and is found on the surface of myocytes and T-lymphocytes (Th0), supporting protective cardiac effects by reducing fibrosis, hypertrophy, and apoptosis [3,22,25]. In contrast, sST2—produced largely by cardiac fibroblasts and endothelial cells under hemodynamic overload, inflammation, and fibrotic stimuli—acts as a decoy receptor that neutralizes IL-33 [24,26]. Consequently, sST2 release dampens the beneficial IL-33/ST2L axis, shifts the immune response from a Th2 (anti-inflammatory) to a Th1 (proinflammatory) profile, and promotes cell death, fibrosis, and progression of heart failure [3,22,25]. Recent evidence indicates that sST2 not only holds prognostic significance, but may also aid in the diagnostic assessment of elevated left ventricular filling pressures when used alongside natriuretic peptides, thereby reinforcing its role as a multifaceted biomarker in heart failure [27].

Our findings align with studies reporting that severe congestion—marked by high CA125 levels—is linked to upregulation of sST2 and other inflammatory pathways [3,9,17]. Elevated CA125 may reflect a state of long-standing tissue fluid overload and inflammatory activation.

### 4.3. Differential Effects of sST2 Across CA125 Levels

Previously, in a post-hoc analysis of the IMPROVE-HF trial [9] involving patients with AHF and renal dysfunction on admission, we found that sST2 was a strong predictor of CV-renal rehospitalizations only when CA125 levels exceeded 35 U/mL. Building on these findings, the present study expands the scope by examining the interaction between sST2 and CA125 in a larger cohort of patients with AHF diagnosed in hospital EDs. Notably, in this broader population, elevated sST2 again proves to be a robust prognostic indicator—specifically, for all-cause mortality and recurrent HF admissions among individuals with CA125 > 35 U/mL.

**Figure 8:**
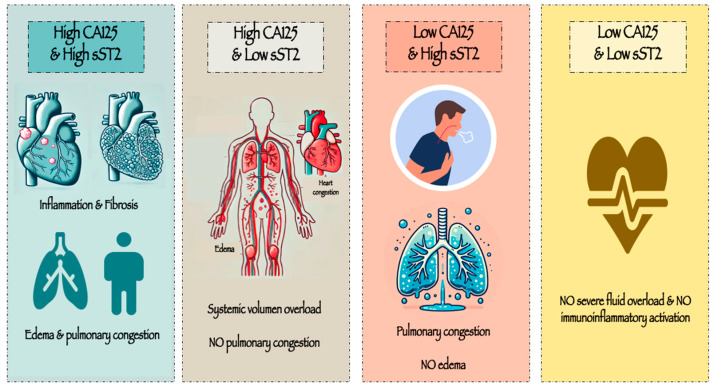
Illustration of the pathophysiological perspective according to CA125 and sST2 levels. The figure was created with the assistance of generative AI.

From a pathophysiological perspective, a concurrent rise in both CA125 and sST2 may indicate sustained tissue congestion together with pronounced inflammation and fibrosis. Conversely, patients whose CA125 exceeds 35 U/mL but present lower sST2 levels might reflect a subgroup characterized primarily by systemic volume overload, without marked pulmonary congestion—an important source of ST2 in heart failure. Meanwhile, patients who exhibit lower CA125 but higher sST2 levels may represent an acute, abrupt onset of AHF, primarily driven by pulmonary intravascular congestion rather than significant interstitial fluid overload. Lastly, those patients with low levels of both biomarkers may be identified as those without severe fluid overload and no immunoinflammatory activation (Figure 8).

One unanswered question is whether the relationship between sST2 and CA125 simply reflects a difference in inflammatory profiles or whether CA125 is mechanistically linked to the sST2 pathway. Further research is needed to clarify whether CA125 exerts a causal influence on sST2 biology or primarily serves as a marker of a distinct pathophysiological state.

The current study has limitations that should be noted. First, the results from this observational analysis must be interpreted only as hypothesis-generating. Second, with the current data, we cannot elucidate the biological mechanisms behind these findings. Third, there are essential unmeasured confounders that have not been assessed and may be playing a role. Fourth, echocardiographic parameters beyond LVEF were not registered. Future studies incorporating more comprehensive echocardiographic assessments to validate our findings and explore their potential clinical implications are required. We cannot extrapolate these findings to chronic HF scenarios.

## 5. Conclusions

Our study highlights the prognostic interplay between sST2 and CA125 in AHF. Elevated sST2 levels predict adverse outcomes primarily in patients with high CA125 levels (>35 U/mL) but not in others. This interaction endorses the role of CA125 as a modulating factor of inflammatory activity in HF. Further studies are warranted.

## Figures and Tables

**Table 1:** Baseline and biomarker characteristics of the total cohort.

	Total Cohort (*n* = 635)
**Demographics and Medical History Data**	
Age, years	82.4 ± 10
Women, *n* (%)	339 (53.4)
Hypertension, *n* (%)	534 (84.1)
Diabetes Mellitus, *n* (%)	248 (39.1)
Dyslipemia, *n* (%)	276 (43.5)
LVEF, %	52.1 ± 13.9
Ischemic heart disease, *n* (%)	161 (25.4)
Valvular heart disease, *n* (%)	189 (29.8)
Chronic kidney disease, *n* (%)	188 (29.6)
Stroke, *n* (%)	82 (12.9)
Prior history of atrial fibrillation, *n* (%)	344 (54.2)
Prior history of HF, *n* (%)	401 (63.1)
COPD, *n* (%)	138 (21.7)
PAD, *n* (%)	59 (9.3)
Barthel index	80.6 ± 24.5
MEESSI Score	−2.8 ± 1.1
Dyspnea, *n* (%)	602 (94.8)
Orthopnea, *n* (%)	341 (53.7)
Pallor, *n* (%)	75 (11.8)
Low output, *n* (%)	97 (15.3)
Peripheral edema, *n* (%)	435 (68.5)
Pleural effusion, *n* (%)	191 (30.1)
Jugular venous distention, *n* (%)	140 (22.0)
Paroxysmal nocturnal dyspnea, *n* (%)	175 (27.6)
Cardiomegaly, *n* (%)	311 (49.0)
Crackles, *n* (%)	422 (66.5)
Third heart sound, *n* (%)	13 (2.0)
NYHA Episode, *n* (%)	
1	6 (0.9)
2	69 (10.9)
3	307 (48.3)
4	253 (39.8)
**Vital signs**	
Heart rate, bpm	87.9 ± 23.7
SBP, mmHg	138.2 ± 26.8
DBP, mmHg	75.8 ± 17.5
Heart rate, bpm	87.9 ± 23.7
**Electrocardiogram**	
Atrial fibrillation, *n* (%)	317 (49.9)
**Laboratory**	
Hemoglobin, g/dL ^1^	12 (10.6–13.4)
Hematocrit (%)	37.3 ± 3.3
Leucocyte count, 10^3^ cells/mL	8914.6 ± 6117.5
Creatinine, mg/dL ^1^	1.2 (0.9–1.6)
Sodium, mEq/L	139.3 ± 4.8
NT-proBNP, pg/mL ^1^	4207 (2280–8421)
CRP, mg/L	23.6 ± 201.8
CA125, U/mL ^1^	44 (19–94)
sST2, ng/mL ^1^	49.2 (27.3–88.5)
CA125 > 35 U/mL, *n* (%)	364 (57.3)
sST2 > 35 ng/mL, *n* (%)	427 (67.2)
**Chronic treatment**	
Beta-blockers, *n* (%)	277 (43.6)
ACEI, *n* (%)	191 (30.1)
ARB, *n* (%)	162 (25.5)
Aldosterone receptor blocker, *n* (%)	95 (15)
Loop diuretics, *n* (%)	419 (66)
Thiazides, *n* (%)	96 (15.1)

^1^ Value expressed as the median (percentile 25–percentile 75). ACEI, Angiotensin-converting enzyme inhibitor; ARB, Angiotensin II receptor blocker; CA125, Antigen Carbohydrate 125; COPD, Chronic Obstructive Pulmonary Disease; CRP, C-reactive protein; DBP; Diastolic blood pressure; HF, heart failure; LVEF, Left ventricular ejection fraction; NT-proBNP, N-terminal pro B-type natriuretic peptide; NYHA, New York Heart Association; PAD, Peripheral Artery Disease; SBP, Systolic blood pressure; sST2, soluble ST2.

**Table 2:** Baseline characteristics according sST2/CA125 categories.

	Gategory 1 CA125 ≤ 35 and sST2 ≤ 35 (*n* = 128)	Category 2 CA125 > 35 and sST2 ≤ 35 (*n* = 80)	Category 3 CA125 ≤ 35 and sST2 > 35 (*n* = 143)	Category 4 CA125 > 35 and sST2 > 35 (*n* = 284)	*p*-Value
**Demographics and medical history**					
Age, years	81.9 ± 9.0	80.0 ± 10.9	84.0 ± 8.6	82.5 ± 10.6	0.029
Women, *n* (%)	83 (64.8)	43 (53.8)	59 (41.3)	154 (54.2)	0.002
Hypertension, *n* (%)	115 (89.8)	69 (86.3)	122 (85.3)	228 (80.3)	0.083
Diabetes Mellitus, *n* (%)	42 (32.8)	23 (28.8)	62 (43.4)	121 (42.6)	0.041
Dyslipemia, *n* (%)	53 (41.4)	32 (40.0)	70 (49.0)	121 (42.6)	0.485
LVEF, %	56.8 ± 13.2	56.5 ± 13.3	51.4 ± 13.9	49.2 ± 13.6	<0.001
Ischemic heart disease, *n* (%)	30 (23.4)	18 (22.5)	40 (28.0)	73 (25.7)	0.770
Valvular heart disease, *n* (%)	40 (31.3)	24 (30.0)	36 (25.2)	89 (31.3)	0.590
Chronic kidney disease, *n* (%)	30 (23.4)	17 (21.3)	47 (32.9)	94 (33.1)	0.060
Stroke, *n* (%)	21 (16.4)	5 (6.3)	22 (15.4)	34 (12)	0.136
Prior history of atrial fibrillation, *n* (%)	73 (57.0)	50 (62.5)	66 (46.2)	155 (54.6)	0.095
Prior history of HF, *n* (%)	89 (69.5)	51 (63.7)	77 (53.8)	184 (64.8)	0.048
COPD, *n* (%)	28 (21.9)	16 (20.0)	35 (24.5)	59 (20.8)	0.819
PAD, *n* (%)	10 (7.8%)	6 (7.5%)	14 (9.8%)	29 (10.2%)	0.810
Barthel index	82.4 ± 23.5	86.6 ± 20.4	82.2 ± 21.4	77.3 ± 26.9	0.011
Dyspnea, *n* (%)	122 (95.3)	79 (98.8)	133 (93.0)	268 (94.4)	0.303
Orthopnea, *n* (%)	61 (47.7)	49 (61.3)	83 (58.0)	148 (52.1)	0.168
Pallor, *n* (%)	12 (9.4)	6 (7.5)	20 (14.0)	37 (13.0)	0.360
Low output, *n* (%)	13 (10.3)	7 (8.8)	29 (20.3)	48 (16.9)	0.039
Peripheral edema, *n* (%)	79 (61.7)	54 (67.5)	101 (70.6)	201 (70.8)	0.290
Pleural effusion, *n* (%)	29 (22.7)	27 (33.8)	34 (23.8)	101 (35.6)	0.014
Jugular venous distention, *n* (%)	20 (15.6)	17 (21.3)	30 (21.0)	73 (25.7)	0.144
Paroxysmal nocturnal dyspnea, *n* (%)	30 (23.4)	29 (36.3)	43 (30.1)	73 (25.7)	0.168
Cardiomegaly, *n* (%)	60 (46.9)	40 (50.0)	63 (44.1)	148 (52.1)	0.430
Crackles, *n* (%)	85 (66.4)	52 (65.0)	91 (63.6)	194 (68.3)	0.795
Third heart sound, *n* (%)	2 (1.6)	2 (2.5)	2 (1.4)	7 (2.5)	0.855
NYHA Episode, *n* (%)					0.013
1	0 (0.0)	0 (0.0)	4 (2.8)	2 (0.7)	
2	15 (11.7)	17 (21.3)	10 (7.0)	27 (9.5)	
3	68 (53.1)	33 (41.3)	66 (46.2)	140 (49.3)	
4	45 (35.2)	30 (37.5)	63 (44.1)	115 (40.5)	
**Vital signs**					
Heart rate, bpm	87.7 ± 23.1	91.3 ± 24.1	86.5 ± 22.5	87.8 ± 24.4	0.542
SBP, mmHg	141.8 ± 26.9	140.2 ± 28.4	141.6 ± 27.3	134.4 ± 25.7	0.012
DBP, mmHg	76.1 ± 15.5	76.2 ± 20	78.6 ± 17.8	74.2 ± 17.3	0.099
**Electrocardiogram**					
Atrial fibrillation, *n* (%)	68 (53.1)	41 (51.2)	65 (45.5)	143 (50.4)	0.627
**Laboratory**					
Hemoglobin, g/dL ^1^	12.3 (11.0–13.6)	11.9 (10.8–13.5)	12.0 (10.7–13.2)	11.8 (10.2–13.4)	0.202
Hematocrit, %	38.2 ± 6.6	37.6 ± 5.7	37.2 ± 5.8	36.9 ± 6.4	0.239
Leucocyte count, 10^3^ cells/mL	8058.3 ± 2804.7	8120.2 ± 3066.0	10,417.3 ± 6827.7	8767.7 ± 7254.2	0.005
Creatinine, mg/dL ^1^	1.1 (0.8–1.3)	1.1 (0.8–1.4)	1.3 (1.0–1.7)	1.2 (0.9–1.7)	<0.001
Sodium, mEq/L	139.6 ± 4.2	139.6 ± 4.1	139.4 ± 4.3	139.0 ± 5.5	0.576
NT-proBNP, pg/mL ^1^	2827.0 (1386.0–4850.0)	4343.0 (1729.5–8207.0)	4380.0 (2484.0–8318.0)	5515.5 (2974.0–11,317.0)	<0.001
CRP, mg/L	15.3 ± 37.0	16.8 ± 39.4	48.4 ± 393.7	14.7 ± 46.2	0.530
**Chronic treatment**					
Beta-blockers, *n* (%)	52 (40.6)	36 (45.0)	55 (38.5)	134 (47.2)	0.315
ACEI, *n* (%)	36 (28.1)	26 (32.5)	45 (31.5)	84 (29.6)	0.892
ARB, *n* (%)	44 (34.4)	17 (21.3)	40 (28.0)	61 (21.5)	0.030
Aldosterone receptor blocker, *n* (%)	16 (12.5)	12 (15.0)	23 (16.1)	44 (15.5)	0.846
Loop diuretics, *n* (%)	83 (64.8)	46 (57.5)	87 (60.8)	203 (71.5)	0.043
Thiazides, *n* (%)	27 (21.1)	15 (18.8)	21 (14.7)	33 (11.6)	0.068

^1^ Value expressed as the median (percentile 25–percentile 75). ACEI, Angiotensin-converting enzyme inhibitor; ARB, Angiotensin II receptor blocker; CA125, Antigen Carbohydrate 125; COPD, Chronic Obstructive Pulmonary Disease; CRP, C-reactive protein; DBP, Diastolic blood pressure; HF, heart failure; LVEF, Left ventricular ejection fraction; NT-proBNP, N-terminal pro B-type natriuretic peptide; NYHA, New York Heart Association; PAD, Peripheral Artery Disease; SBP, Systolic blood pressure; sST2, soluble ST2.

## Data Availability

Data are unavailable due to privacy or ethical restrictions.

## Abbreviations

AHF	Acute heart failure
ANOVA	Analysis of variance
CA125	Antigen carbohydrate 125
CTD	C-terminal domain
CV	Cardiovascular
EAHFE	Epidemiology of Acute Heart Failure in Spanish Emergency Departments
EDs	Emergency departments
EMT	Epithelial-to-mesenchymal transition
Gal-1	Galectin-1
HF	Heart failure
IL-33	Interleukin-33
IQR	Interquartile range
JNK	c-Jun N-terminal kinase
LVEF	Left ventricular ejection fraction
MUC16	Mucin 16
NYHA	New York Heart Association
P-Y	Person-years
sST2	Soluble Suppression of Tumorigenicity 2
ST2L	Membrane-bound ST2
Th0	T-lymphocytes
TNF	Tumor necrosis factor

## Appendix A

**Table A1:** Death risk estimates for covariates included in the final prognostic multivariate models.

Variable	HR	CI	*p*-Value
Age, per ten-year increase	1.20	1.01–1.43	0.046
Men	1.30	0.96–1.77	0.087
Prior known HF	1.28	0.93–1.53	0.184
NYHA class prior to admission, per increase in 1 category	1.10	0.94–1.27	0.185
Atrial fibrillation	1.23	0.96–1.76	0.159
Ischemic heart disease	1.28	0.93–1.78	0.133
Barthel index, per increase of 1 point	0.98	0.98–0.99	<0.001
Heart rate, per increase of 1 bpm	1.02	1.01–1.04	0.031
SBP, per increase of 10 mmHg	0.95	0.89–0.99	0.041
Hemoglobin, per increase of 1 g/dL	0.95	0.89–1.01	0.149
eGFR, per increase of 10 mL/min/1.73 m^2^	0.95	0.92–0.98	0.002
Orthopnea	1.24	0.93–1.56	0.195
Crackles	0.90	0.67–1.22	0.493
Paroxysmal nocturnal dyspnea	0.95	0.67–1.34	0.760
Third heart sound	1.99	0.90–4.44	0.092
Jugular venous distension	1.12	0.80–1.59	0.515
Pleural effusion	1.10	0.80–1.50	0.534
Peripheral edemas	0.80	0.59–1.10	0.176

**Table A2:** Death or HF admission risk estimates for covariates included in the final prognostic multivariate models.

Variable	HR	CI	*p*-Value
Age, per ten-year increase	1.18	1.01–1.37	0.038
Men	1.19	0.92–1.54	0.190
Prior known HF	1.23	0.94–1.61	0.126
NYHA class prior to admission, per increase in 1 category	1.36	0.95–1.87	0.120
Atrial fibrillation	0.91	0.70–1.15	0.430
Ischemic heart disease	1.39	1.05–1.83	0.023
Barthel index, per increase of 1 point	0.99	0.98–0.99	0.001
Heart rate, per increase of 1 bpm	1.00	1.00–1.01	0.161
SBP, per increase of 10 mmHg	0.95	0.91–0.99	0.046
Hemoglobin, per increase of 1 g/dL	0.94	0.89–1.00	0.037
eGFR, per increase of 10 mL/min/1.73 m^2^	0.98	0.95–0.99	0.045
Orthopnea	1.15	0.88–1.49	0.300
Crackles	1.00	0.77–1.30	0.995
Paroxysmal nocturnal dyspnea	0.87	0.65–1.17	0.363
Third heart sound	1.19	0.53–2.65	0.675
Jugular venous distension	1.07	0.80–1.43	0.671
Pleural effusion	1.10	0.85–1.43	0.471
Peripheral edemas	0.99	0.76–1.30	0.962
